# AldoC BAC-GFP transgenic mice as a reliable model for astrocyte identification and functional studies in the brain

**DOI:** 10.1186/s13041-025-01264-0

**Published:** 2025-12-04

**Authors:** Juhyun Kim, Hayoung Yang, Seong Seop Kim, Eunsil Cho, Song Her, Eun Mi Hwang, Sungbo Shim, Jae-Yong Park

**Affiliations:** 1 Department of Integrated Biomedical and Life Sciences, Korea University, Seoul, 02841 Korea; 2 BK21FOUR R&E Center for Learning Health Systems, Korea University, Seoul, 02841 Korea; 3 Department of Biochemistry, Chungbuk National University, Cheongju, 28644 Korea; 4 Metropolitan Seoul Center, Korea Basic Science Institute, Seoul, 02841 Korea; 5 Brain Science Institute, Korea Institute of Science and Technology (KIST), Seoul, 02792 Korea

**Keywords:** Aldolase C, Bacterial artificial chromosome transgenic mouse, Green fluorescent protein, Astrocyte marker, Passive conductance

## Abstract

Astrocytes, the most abundant glial cell type in the central nervous system (CNS), are essential for maintaining neural homeostasis, forming gliovascular unit, and modulating synaptic function. However, commonly used astrocytic markers often display regional variability or lack strict specificity, limiting their reliability for consistently identifying astrocytes across brain regions. To address this limitation, we generated a novel transgenic mouse line (AldoC BAC-GFP) that expresses green fluorescent protein (GFP) under the control of the aldolase C (AldoC) promoter using modified bacterial artificial chromosome (BAC) technology. AldoC is an enzyme abundantly expressed in astrocytes. We confirmed that GFP-expressing cells in these mice co-express endogenous AldoC and are co-labeled with established astrocytic markers, thereby validating their astrocytic identity. Importantly, GFP expression was largely restricted to astrocytes throughout diverse brain regions. Moreover, GFP-positive astrocytes in brain slices exhibited the characteristic linear-shaped passive conductance of mature astrocytes. Collectively, these findings demonstrate that AldoC BAC-GFP transgenic mice represent a reliable and broadly applicable model for functional studies of astrocytes in the CNS.

**Supplementary Information:**

The online version contains supplementary material available at 10.1186/s13041-025-01264-0.

## Introduction

Astrocytes, a major glial cell types in the central nervous system (CNS), constitute a substantial portion of the brain’s cellular composition. Traditionally regarded as passive supporting elements, astrocytes are now recognized for their diverse functions, including maintaining structural integrity [1], supplying metabolic substrates [2], forming a specialized gliovascular unit [3] and regulating neuronal ionic homeostasis [4–6]. Beyond these supportive roles, accumulating evidences demonstrate that astrocytes actively participate in synaptic transmission [7, 8], modulate neural circuits [9], and regulate brain plasticity [10, 11], underscoring their pivotal contributions to both normal brain functions and neurological disorders.

Astrocytes are widely distributed throughout the brain and display region-specific morphological and molecular diversity [12]. Recent studies using regional proteomic profiling have shown that astrocytes in distinct brain regions express unique protein signatures and perform specialized functions [13]. Accordingly, precise and reliable identification of astrocytes has become increasingly important for advancing astrocyte research. Several markers—including glial fibrillary acidic protein (GFAP), S100β, glutamine synthetase (GS), glutamate transporter 1 (GLT-1), and aldehyde dehydrogenase 1 family member L1 (Aldh1l1)—have been widely used. However, each has critical limitation in specificity or consistency. For instance, subpopulation of GFAP-negative astrocytes is nearly 40% of astrocytes in adult CA1 molecular layer and GFAP-positive astrocytes are less found in thalamus [14–16]. S100β colocalizes with the microglial marker Iba1 in the cortex [17], while GS and GLT-1 are also expressed in oligodendrocytes [18]. Similarly, Aldh1l1 is expressed in neural stem cells and often produces diffuse labeling, complicating morphological analyses [19]. These limitations highlight the urgent need for a more specific and broadly applicable tool to consistently identify astrocytes across brain regions.

To address this gap, we developed a novel AldoC BAC-GFP transgenic mouse line, in which green fluorescent protein (GFP) is expressed under the control of the astrocyte-enriched aldolase C (AldoC) promoter. Aldolase C, a glycolytic enzyme catalyzing the reversible cleavage of fructose-1,6-bisphosphate, is abundantly expressed in the CNS, particularly in astrocytes, where it contributes to astrocyte-specific metabolic functions [13, 20]. Importantly, AldoC exhibits high and consistent expression across diverse CNS regions, supporting its utility as a reliable pan-astrocytic marker. In the present study, we generated AldoC BAC-GFP transgenic mice using bacterial artificial chromosome (BAC) technology, thereby preserving the endogenous regulatory elements of the AldoC gene. This strategy enables physiologically relevant GFP expression in AldoC-expressing cells. With this model, we sought to (1) validate the astrocyte specificity of AldoC expression, (2) evaluate its utility as a brain-wide astrocyte marker, and (3) assess the applicability of AldoC BAC-GFP transgenic mice as a reliable tool for functional studies of astrocytes.

## Materials and methods

### Generation and maintenance of transgenic mice

A modified bacterial artificial chromosome (BAC) clone encompassing the *Aldolase C* (*AldoC*) locus (GENSAT1-BX1126; Entrez Gene ID: 11,676) was obtained from the BACPAC Resources Center. This clone was derived via recombination from the parental BAC RP24-353H15, as described on the GENSAT website (www.gensat.org). BAC DNA was purified using a Large-Construct Kit (Qiagen, Cat# 12,462). Pronuclear injection was performed using fertilized eggs from C57BL/6N females, and the embryos were transplanted into pseudopregnant ICR females (Orient Bio, Seongnam-si, Korea). Transgenic AldoC BAC-GFP mice were genotyped using the following primers: forward 5′-GGAACTGGGGCCCTAACTC-3′ and reverse 5′-ATGTGATCGCGCTTCTCGTT-3′. Male transgenic mice at postnatal day 56 (P56) were used for subsequent experiments, based on AldoC mRNA expression data from the Allen Brain Atlas (https://mouse.brain-map.org/gene/show/11463). All animal procedures were approved by the Korea University Institutional Animal Care and Use Committee (approval number: KU-IACUC-2022–0024).

### Western blot

Forebrain tissues from male AldoC BAC-GFP transgenic and wild-type mice (7–9 weeks old; N_TG_ = 3, N_WT_ = 3) were lysed in RIPA buffer (T&I) supplemented with a protease inhibitor cocktail (Roche). Protein lysates (20 μg per lane) were resolved by 10% SDS-PAGE and transferred to PVDF membranes. Membranes were blocked in 5% non-fat dry milk and incubated overnight at 4 °C with the following primary antibodies: rabbit anti-AldoC (Abcam, ab190368, 1:1000), rabbit anti-GFP (GeneTex, GTX26673, 1:1000), mouse anti-β-tubulin (Santa Cruz Biotechnology, sc-166729, 1:1000), and mouse anti-β-actin (Sigma-Aldrich, A5441, 1:1000). After washing, membranes were incubated for 1 h at RT with HRP-conjugated secondary antibodies: rabbit anti-mouse IgG (Bethyl Laboratories, A90-117P, 1:5000), goat anti-rabbit IgG (A120-101P, 1:5000), or donkey anti-goat IgG (A50-101P, 1:5000). Immunoreactive bands were visualized using enhanced chemiluminescence (ECL; BIO-RAD, 1,705,061).

### Quantitative real-time PCR

Total RNA was extracted from mouse brain tissues using an RNA purification kit (GeneAll, 305–101), and concentration and purity were determined via spectrophotometry at 260/280 nm. Reverse transcription was carried out using 1 μg of RNA and the RNA to cDNA EcoDry™ Premix (Takara, 639,543) according to the manufacturer’s protocol. Quantitative PCR was performed using SYBR Green Master Mix (Enzo, ENZ-NUC104-1000) and the following primer pairs: *AldoC*: F 5′–ATCCAATGCCTGGAGAGGAC–3′; R 5′–CGATGTAGAGGGACTGTGCT–3′, *GFP*: F 5′–GCCACAAGTTCAGCGTGTCC–3′; R 5′–TAGCGGCTGAAGCACTGCA–3′, *GAPDH* (reference): F 5′–AATGTGTCCGTCGTGGATCT–3′; R 5′–AGACAACCTGGTCCTCAGTG–3′.

Relative expression was calculated using the 2^−ΔΔCt^ method, and all reactions were performed in triplicate from at least three independent biological replicates.

### Brain slice preparation

Male AldoC BAC-GFP transgenic and wild-type mice (7–9 weeks old; N_TG_ = 3, N_WT_ = 3) were anesthetized with chloroform and perfused transcardially with PBS followed by 4% paraformaldehyde (PFA). Brains were post-fixed overnight in 4% PFA, cryoprotected in 30% sucrose for 72 h, embedded in OCT compound (Sakura Finetek, Cat# 4583), and frozen. Coronal Sects. (30 µm) were prepared using a cryostat (Leica).

### Immunohistochemistry

Brain Sects. (30 μm) were washed three times with PBS (10 min each) at room temperature (RT), followed by antigen retrieval in 10 mM sodium citrate buffer (pH 6.0) at 85 °C for 30 min. After cooling, slices were rinsed with PBS and permeabilized with 0.4% Triton X-100 in PBS for 35 min at RT. Non-specific binding was blocked using 10% donkey serum and 0.1% Triton X-100 in PBS for 3 h at RT, followed by overnight incubation at 4 °C with primary antibodies diluted in PBS containing 5% donkey serum and 0.1% Triton X-100.

The following primary antibodies were used: mouse anti-AldoC (Abcam, ab190368, 1:1000), mouse anti-glutamine synthetase (Millipore, MAB302, 1:500), chicken anti-GFAP (Invitrogen, PA1-10,004, 1:500), mouse anti-S100β (Sigma-Aldrich, S2532, 1:500), guinea pig anti-GLT-1 (Millipore, AB1783, 1:500), rabbit anti-Iba1 (Wako, 019–19741, 1:2000), mouse anti-NeuN (Abcam, ab104224, 1:500), rabbit anti-Calbindin (Swant, CB38, 1:2000), rabbit anti-Sox9 (ABclonal, A19710, 1:500), rabbit anti-Aldh1l1 (Abcam, ab87117, 1:300), and rabbit anti-TREK-1 (Alomone labs, APC-047, 1:500). After washing with 0.1% Triton X-100 in PBS (3 × 10 min), sections were incubated for 90 min at 4 °C with species-appropriate secondary antibodies conjugated to Alexa Fluor dyes: Alexa Fluor 488-, 594-, or 647-conjugated donkey anti-rabbit, anti-mouse, or anti-goat IgG (Jackson ImmunoResearch, all 1:300). Nuclei were counterstained with DAPI and sections were mounted using VECTASHIELD antifade mounting medium (Vector Laboratories, H-1000). Macroscopic fluorescence images were acquired using a ZEISS Axio Scan.Z1 slide scanner (RRID: SCR_020927), and high-resolution confocal images were obtained using a Nikon Eclipse Ti2 microscope (RRID: SCR_021068).

### Electrophysiological recordings in hippocampal slices

Acute hippocampal slices (350 µm) were prepared from male AldoC BAC-GFP transgenic and wild-type mice (7–9 weeks old; N_TG_ = 3, N_WT_ = 3) using a Leica VT1000S vibratome. Slices were recovered in oxygenated (95% O_2_ / 5% CO_2_) artificial cerebrospinal fluid (ACSF: 130 mM NaCl, 24 mM NaHCO_3_, 3.5 mM KCl, 1.25 mM NaH_2_PO_4_, 1.5 mM CaCl_2_, 1.5 mM MgCl_2_, and 10 mM glucose, pH 7.4) for 1 h. Astrocytes in wild-type slices were labeled with SR-101 (1 µM, Sigma) for 30 min prior to recordings. Whole-cell patch-clamp recordings were conducted using Axopatch 200A (Axon Instruments) with intracellular solution containing: 140 mM KCl, 10 mM HEPES, 5 mM EGTA, 2 mM Mg-ATP, and 0.2 mM Na-GTP (pH 7.4). Currents were recorded with 1 s voltage steps from − 160 mV to + 40 mV in 10 mV increments from a holding potential of − 60 mV.

### Statistical analysis

All results are expressed as mean ± standard error of the mean (SEM). Statistical significance was evaluated using unpaired Student’s *t*-tests or one-way ANOVA followed by Tukey’s post hoc test. Significance thresholds were set as follows: n.s., not significant; *p < 0.05; **p < 0.01; ***p < 0.001; ****p < 0.0001. GraphPad Prism 9.0 (GraphPad Software) was used for data analysis.

## Results

### Generation of AldoC BAC-GFP transgenic mice

Aldolase C (AldoC) is predominantly expressed in astrocytes and shows strong expression across multiple brain regions [13]. Owing to its regional consistency and cell-type specificity, AldoC was selected as a candidate for developing a universal astrocytic marker. Western blot analysis revealed that AldoC expression was more uniformly distributed across brain regions compared to established markers such as GFAP and GLT-1 (Fig. S1A–B). Immunohistochemistry (IHC) with an anti-AldoC antibody further confirmed its broad distribution throughout the brain (Fig. S1C). These findings supported the rationale for selecting AldoC as a representative astrocytic marker.

**Fig. 1:**
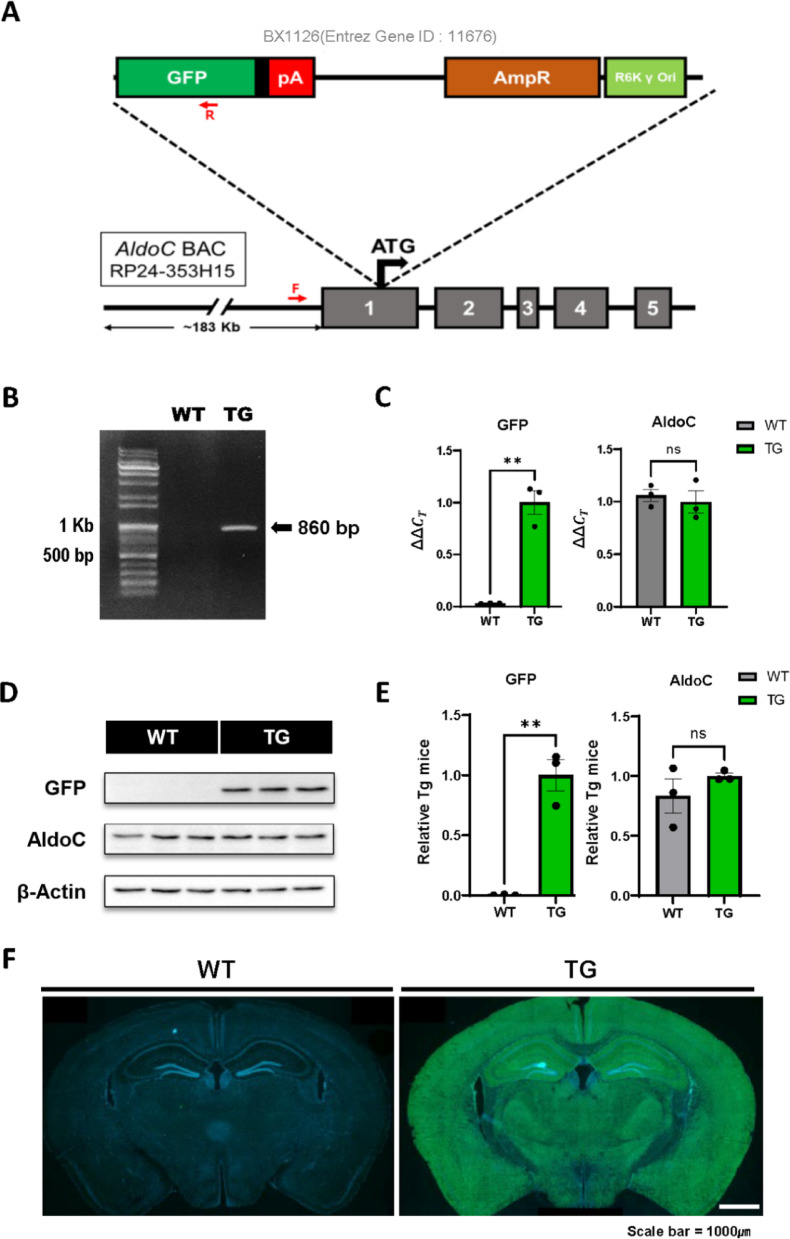
Generation of AldoC BAC-GFP transgenic mice. **A** Schematic illustration of the BAC construct driving GFP expression under the control of the *Aldolase C (AldoC)* promoter. **B** Genotyping PCR to distinguish transgenic (TG; 860 bp) from wild-type (WT) alleles using genomic DNA. **C** Quantitative RT-PCR analysis showing comparable *AldoC* mRNA levels between TG and WT mice. (N_TG_ = 3, N_WT_ = 3). **D**, **E** Western blot analysis of AldoC protein expression in brain tissue from TG and WT mice; β-actin was used as a loading control. (N_TG_ = 3, N_WT_ = 3). **F** Macroscopic fluorescence images were acquired using a slide scanner. GFP fluorescence confirming expression in AldoC BAC-GFP transgenic mice but not in WT controls. Scale bars: 1000 µm

To visualize AldoC expression in vivo, we generated AldoC BAC-GFP transgenic (Tg) mice using a modified BAC clone (RP24-353H15) containing the AldoC promoter and regulatory elements (Fig. 1A). A GFP cassette was inserted into the BAC construct, and transgenic founders were identified by PCR genotyping (Fig. 1B). PCR analysis confirmed that endogenous AldoC expression was comparable between wild-type (WT) and Tg mice (Fig. 1C), and Western blotting further validated that AldoC protein levels were unaltered in Tg mice (Fig. 1D–E). Robust GFP fluorescence was observed in brain tissues from Tg mice (Fig. 1F).

### Validation of AldoC BAC-GFP expression across brain regions

**Fig. 2:**
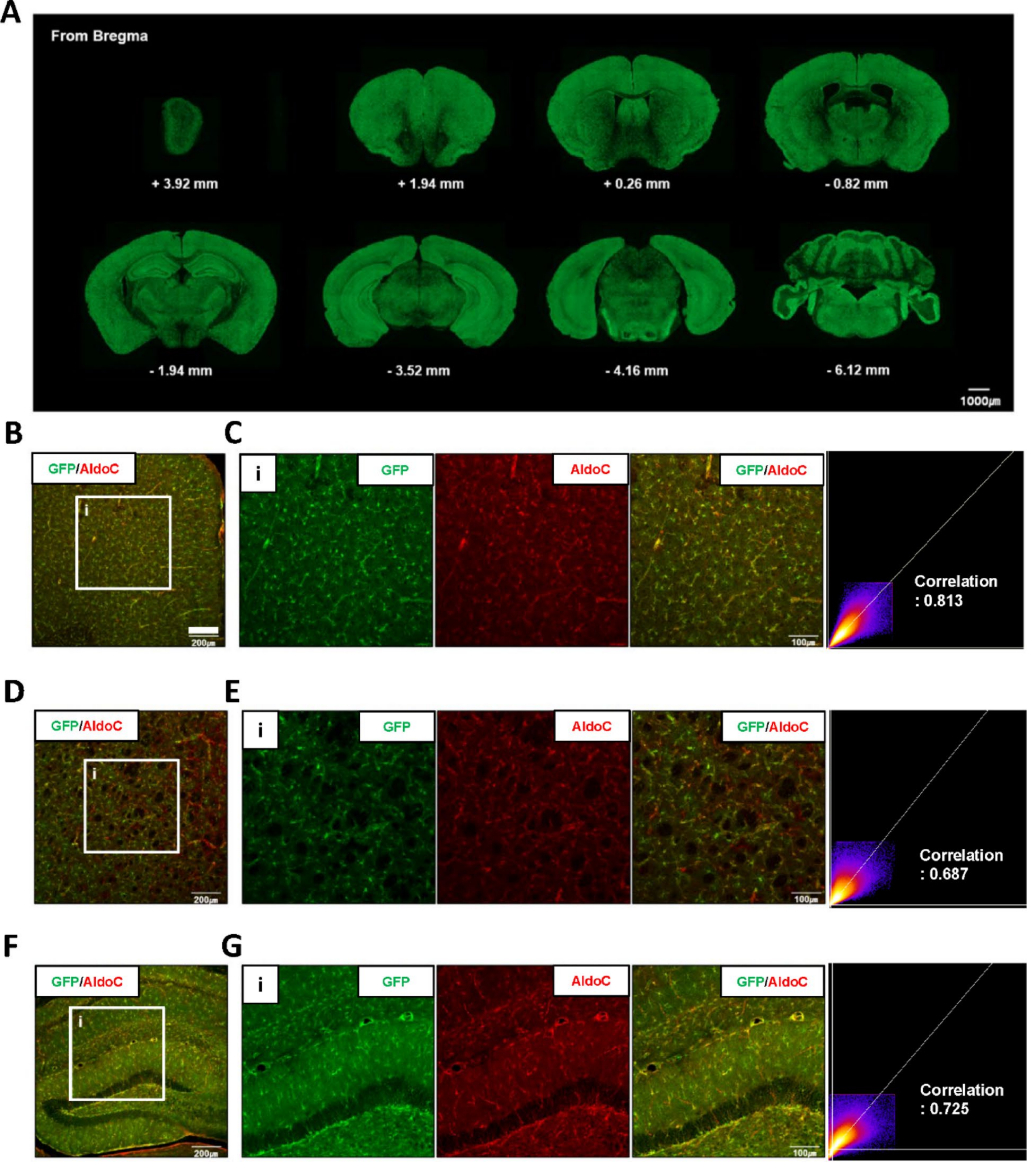
Region-wide expression of GFP in the brain of AldoC BAC-GFP mice. **A** Whole-brain sagittal view of GFP expression in AldoC BAC-GFP mice. Fluorescence images were acquired using a slide scanner. Scale bar: 1000 µm. (B–C) Representative co-immunofluorescence images showing colocalization of GFP and AldoC in the prefrontal cortex. **B** Scale bars: 200 µm, **C** 100 µm. (D–E) Colocalization of GFP and AldoC in the striatum. **D** Scale bars: 200 µm, **E** 100 µm. (F–G) Colocalization of GFP and AldoC in the hippocampus. **F** Scale bars: 200 µm, **G** 100 µm. Colocalization images of GFP and AldoC were acquired using a confocal microscope

IHC of sagittal brain sections revealed that GFP fluorescence in AldoC BAC-GFP mice mirrored the endogenous AldoC expression pattern and was widely distributed across brain regions (Fig. 2A). Colocalization analysis confirmed that GFP-expressing cells in the prefrontal cortex, striatum, and hippocampus co-expressed AldoC protein (Fig. 2B–G). In the cerebellum, GFP fluorescence also overlapped with AldoC, as well as with calbindin, indicating expression in Purkinje cells (Fig. S2A–D). Region-specific variation in GFP-positive cell density and morphology was observed. High GFP expression was found in the cortex and olfactory bulb, while lower expression was observed in the corpus callosum, superior colliculus (zonal layer), and pons. GFP-positive cells exhibited radial morphologies in the cortex and olfactory bulb, whereas linear morphologies were evident in the cerebellum and pons (Fig. S3). These results suggest that the AldoC BAC-GFP model allows for comparative analysis of astrocyte morphology and density across brain regions.

### Astrocytic specificity of GFP expression in AldoC BAC-GFP mice

**Fig. 3:**
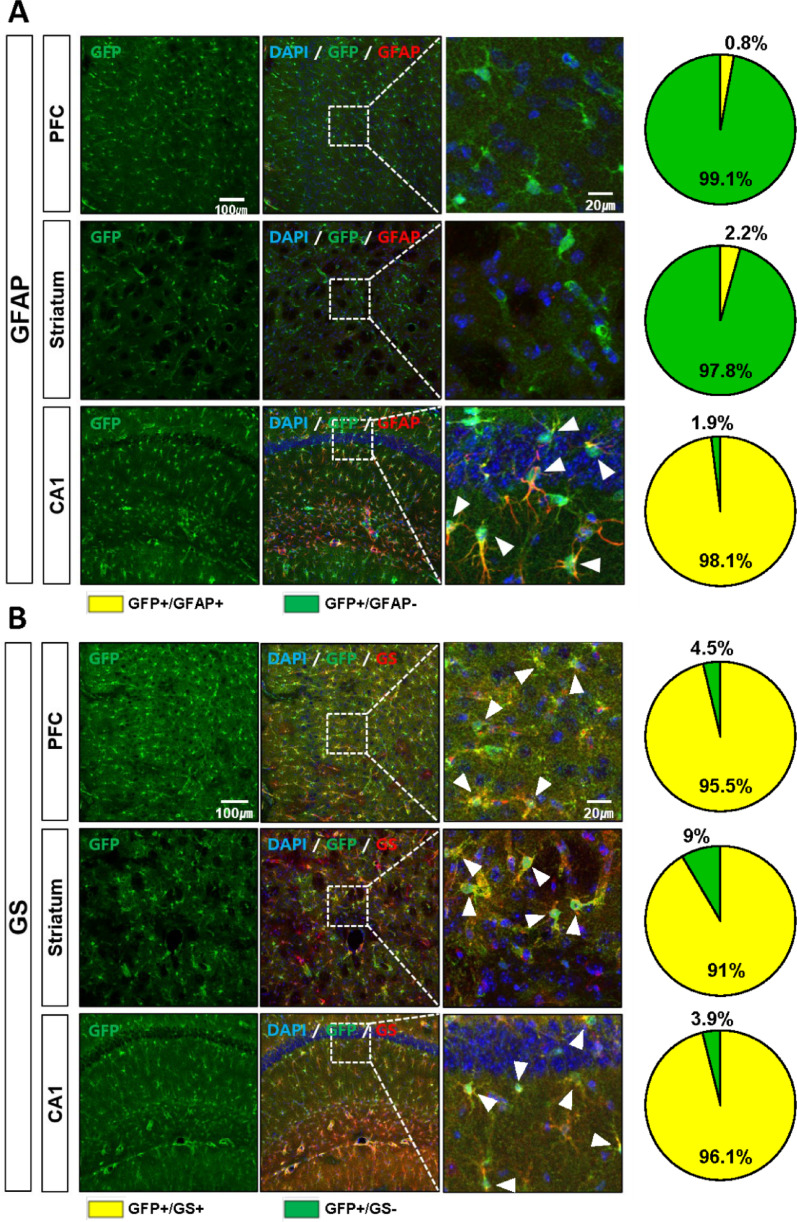
GFP-expressing cells co-localize with astrocytic markers GFAP and GS. **A**, **B** Representative confocal images and quantification pie charts showing colocalization of GFP with GFAP (at least 830 cells were counted) and glutamine synthetase (GS) (at least 742 cells were counted) in the prefrontal cortex (PFC), striatum, and hippocampal CA1. Scale bars: 100 µm (overview), 20 µm (zoom). Arrowheads indicate double-labeled astrocytes. (N_TG_ = 3, N_WT_ = 3). All images were acquired using a confocal microscope

**Fig. 4:**
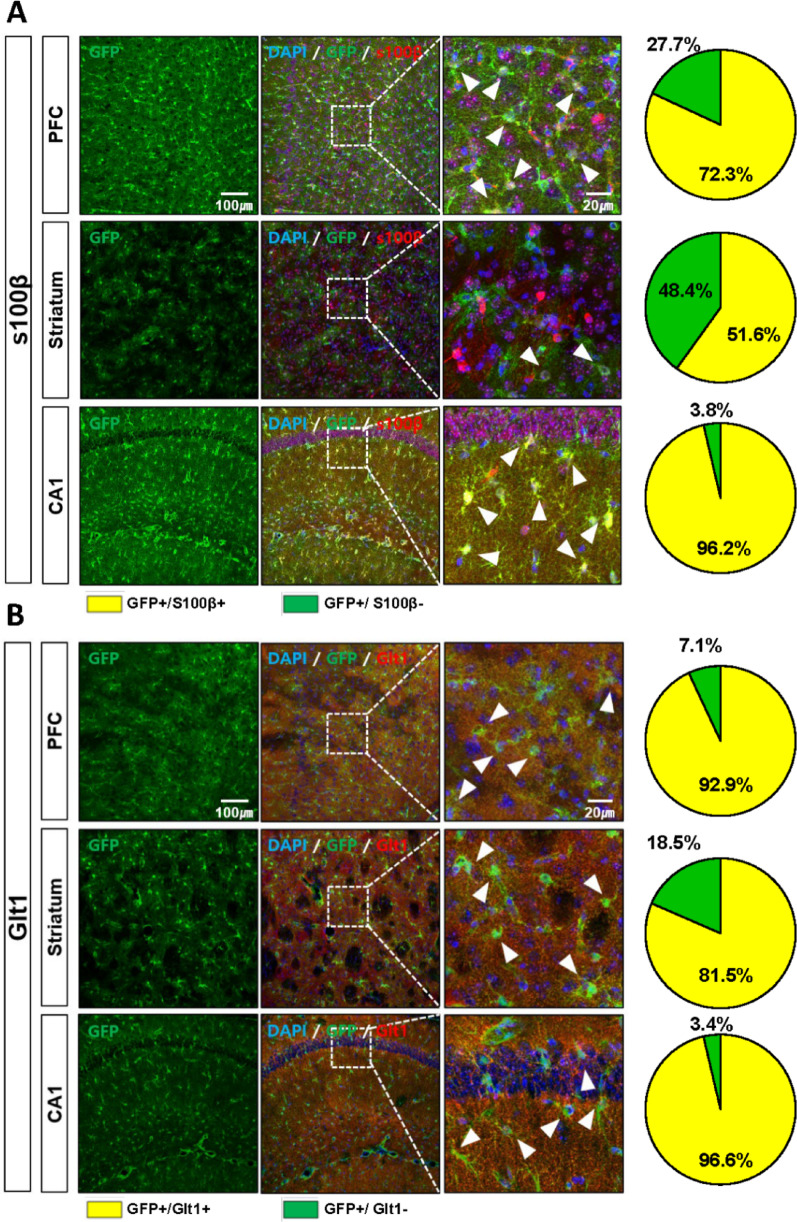
Partial overlap of GFP with astrocytic markers S100β and GLT-1. **A**, **B** Representative images and quantification showing regional differences in colocalization of GFP with S100β and GLT-1 in the PFC, striatum, and CA1. Scale bar: 20 µm. Arrowheads indicate double-positive cells. (N_TG_ = 3 (*n* = *6*), N_WT_ = 3 (*n* = *6*)). All images were acquired using a confocal microscope

To determine the cellular identity of GFP-positive cells, we assessed their colocalization with cell-type-specific markers across multiple CNS regions, including the prefrontal cortex, striatum, and CA1 region of the hippocampus (Fig. S3). GFP signals showed strong overlap with astrocytic markers such as GFAP, glutamine synthetase (GS), S100β, and GLT-1. In GFAP-low regions (prefrontal cortex and striatum), colocalization with GFAP was minimal (0.8% and 2.2%, respectively), whereas in CA1, a GFAP-rich region, colocalization reached 98.1% (Fig. 3A). By contrast, colocalization with GS was consistently high across all regions examined—95.5% in the prefrontal cortex, 91.0% in the striatum, and 96.1% in CA1 (Fig. 3B). S100β and GLT-1 exhibited more variable patterns: S100β colocalized in 72.3% (prefrontal cortex), 51.6% (striatum), and 96.2% (CA1), while GLT-1 colocalized in 92.9, 81.5 and 96.6% of GFP-positive cells in the same regions (Fig. 4A–B).

We also examined the colocalization of GFP-positive cells with other widely used astrocytic markers, Aldh1L1 and Sox9 (Fig. S4). IHC revealed that over 90% of Aldh1L1-positive signals overlapped with GFP-positive cells in the prefrontal cortex (PFC), striatum, and CA1 regions. In contrast, more than 70% of Sox9-positive signals overlapped with GFP-positive cells across all regions; however, this overlap exceeded 90% only in the CA1 region (Fig. S4A–B). These findings indicate that while Aldh1L1 predominantly labels mature astrocytes, Sox9 marks a broader cell population, including radial glial cells (RGCs) [21].

**Fig. 5:**
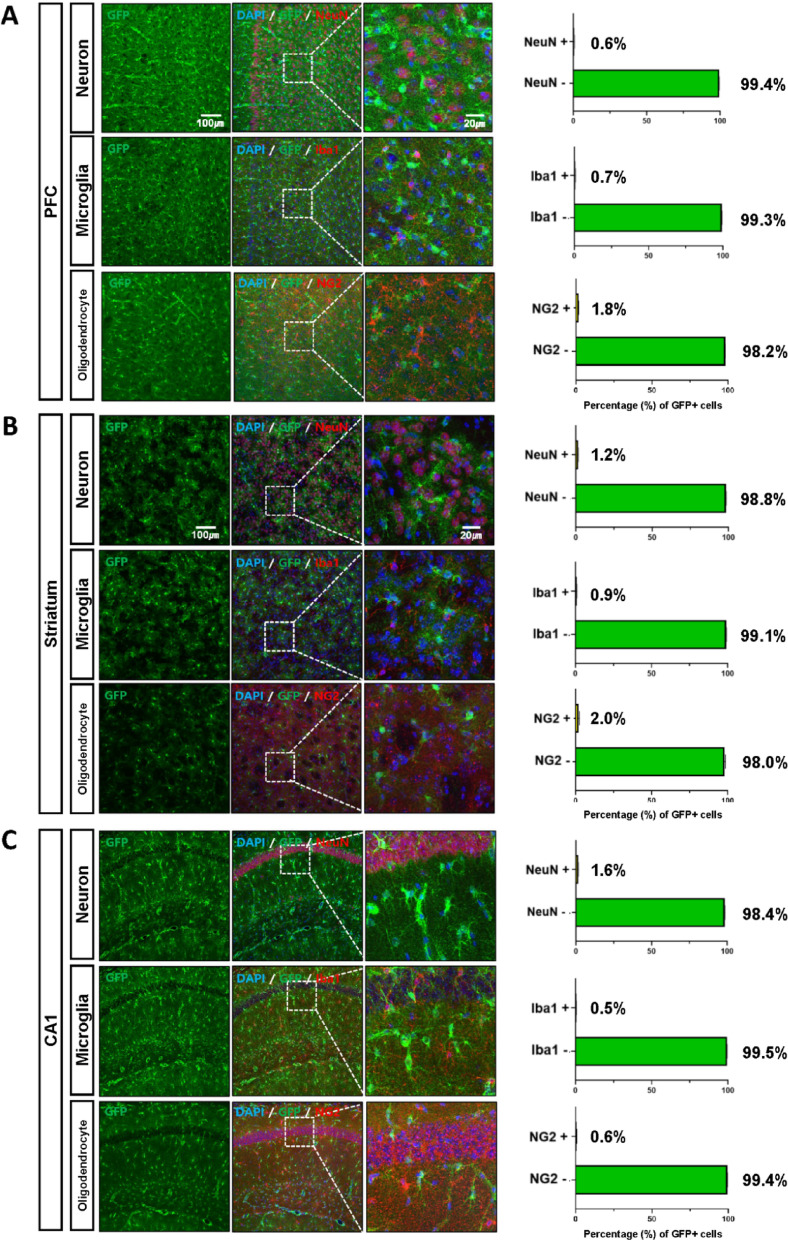
GFP-expressing cells do not colocalize with neuronal, microglial, or oligodendrocyte markers. **A**–**C** Representative confocal images and quantification showing minimal colocalization of GFP with NeuN (neuronal marker), Iba1 (microglial marker), and NG2 (oligodendrocyte precursor marker) in the PFC, striatum, and CA1 regions. (N_TG_ = 3 (*n* = *6*), N_WT_ = 3 (*n* = *6*)). All images were acquired using a confocal microscope

In contrast, GFP-positive cells showed minimal overlap with non-astrocytic markers, including NeuN (neuron), Iba1 (microglia), and NG2 (oligodendrocyte precursor), with ≤ 2% colocalization across all regions tested (Fig. 5A–C). Co-expression of GS and NG2 or GLT-1 and NG2 was also confirmed in the hippocampus (Fig. S5A–B). Together, these results demonstrate that GFP-expressing cells in AldoC BAC-GFP mice represent astrocytes with high specificity.

### Astrocytic heterogeneity in the habenula

**Fig. 6:**
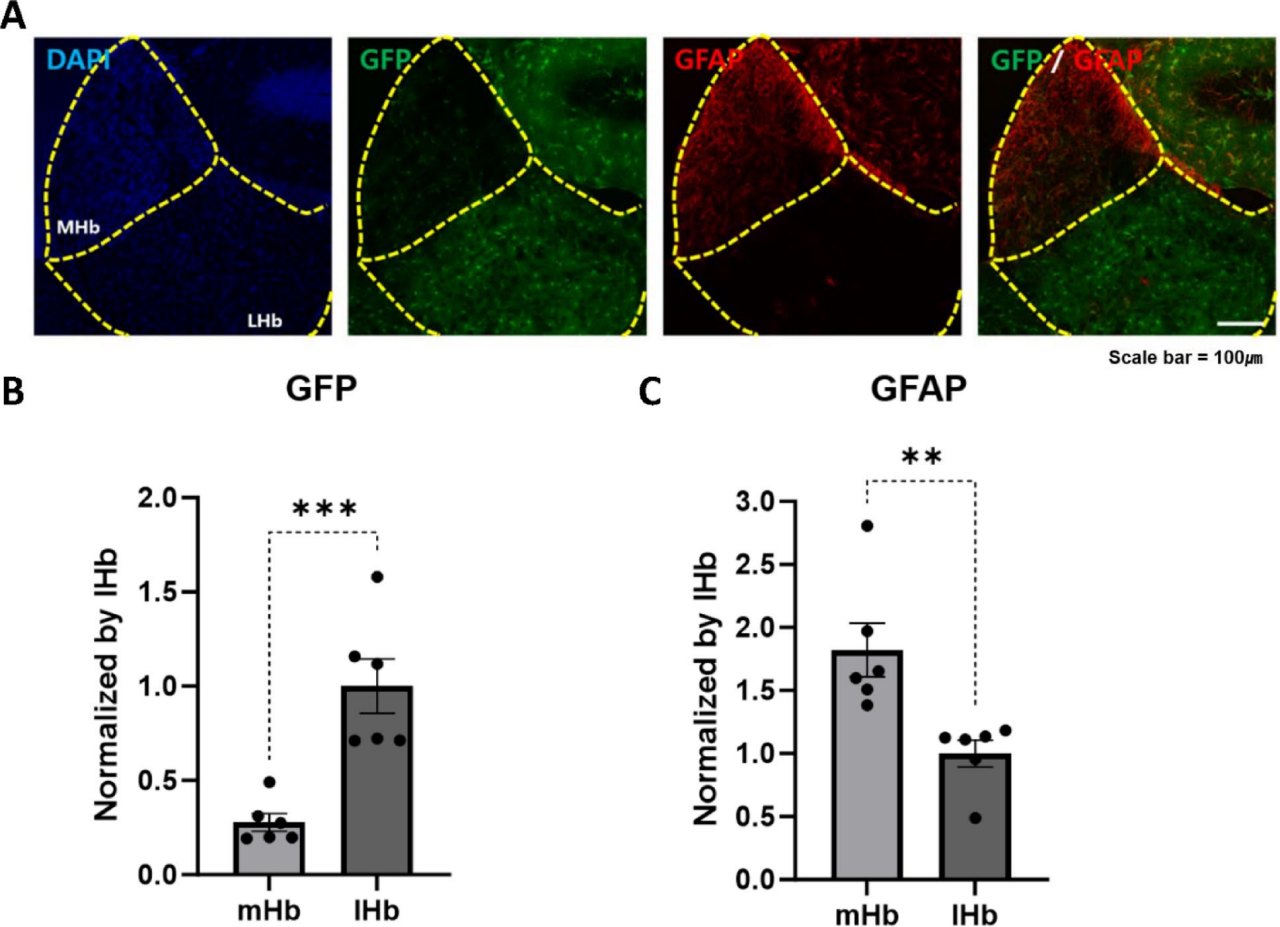
Differential distribution of GFP-expressing astrocytes in the medial and lateral habenula. **A** Representative images of GFP expression and GFAP immunostaining in the medial (mHb) and lateral habenula (lHb). Images were acquired using a confocal microscope. **B** Quantification of GFP-positive cell density in each subregion. Scale bar: 100 µm. (C) GFAP expression was observed primarily in the mHb, whereas AldoC-GFP expression was enriched in the lHb. (N_TG_ = 3 (*n* = *6*), N_WT_ = 3 (*n* = *6*))

Interestingly, although AldoC BAC-driven GFP expression was evident in most brain regions, no detectable GFP fluorescence was observed in the medial habenula (mHb) (Fig. 1F, Fig. 2A). To further explore astrocytic heterogeneity within the habenula, we compared marker expression between the mHb and lateral habenula (lHb). Immunostaining revealed that GS and GLT-1 were more enriched in the lHb, whereas S100β was more prominent in the mHb (Fig. S6A–F). Consistently, GFP signal was robust in the lHb but absent in the mHb (Fig. 6A–B), while GFAP expression was restricted to the mHb (Fig. 6A, C). Together, these findings highlight the spatial segregation of astrocyte subtypes in the habenula, with AldoC-expressing astrocytes predominating in the lHb and GFAP-positive astrocytes localized to the mHb.

### Electrophysiological validation of GFP-positive astrocytes in AldoC BAC-GFP mice

**Fig. 7:**
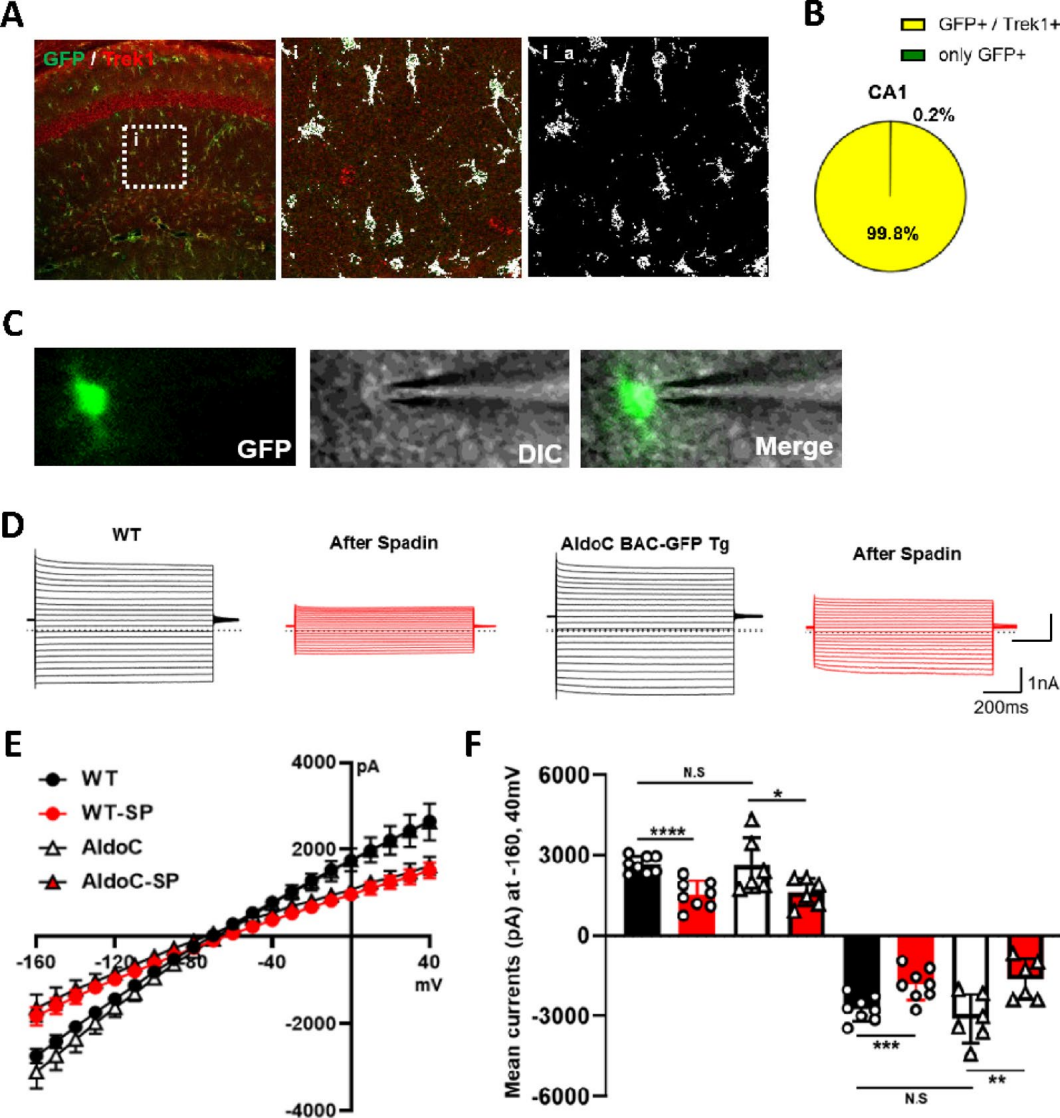
GFP-positive astrocytes in AldoC BAC-GFP mice exhibit passive conductance. **A**, **B** Co-immunostaining and quantification showing colocalization of GFP with TREK-1 in hippocampal CA1 astrocytes. Images were acquired using a confocal microscope. **C** Image of a patch-clamp setup targeting a GFP-positive astrocyte in hippocampal slice. Images were acquired using CCD camera of slice-patch setup. **D** Representative whole-cell current traces recorded in astrocytes from WT and AldoC BAC-GFP mice, with and without Spadin treatment (a TREK-1 inhibitor). **E** I–V curves from recordings in (D), showing linear passive conductance suppressed by Spadin. **F** Bar graph summarizing passive conductance amplitudes. Data are presented as mean ± SEM; statistical significance determined by Student’s *t*-test: n.s., not significant; *p < 0.05; **p < 0.01;*** *p* < 0.001. (N_TG_ = 3 (*n* = *8*), N_WT_ = 3 (*n* = *8*))

Astrocytes are characterized by passive, linear potassium currents that distinguish them neuronal electrical activity. To determine whether GFP-positive cells in AldoC BAC-GFP mice functionally represent astrocytes, we performed whole-cell patch-clamp recordings. Immunohistochemistry confirmed that TREK-1, a potassium channel mediating passive conductance, was expressed in 99.8% of GFP-positive hippocampal cells (Fig. 7A–B) [22]. Without the use of additional dyes, GFP fluorescence reliably identified astrocytes for recording (Fig. 7C). In wild-type hippocampal slices, application of Spadin, a TREK-1 inhibitor, reduced passive conductance, confirming the functional contribution of TREK-1 (Fig. 7D) [23, 24]. Consistent with this, GFP-positive cells in AldoC BAC-GFP mice exhibited linear passive currents that were abolished upon Spadin treatment (Fig. 7D–F). Together, these results demonstrate that GFP-expressing cells in AldoC BAC-GFP mice display canonical astrocytic electrophysiological properties, validating the utility of this model for functional studies.

## Discussion

In this study, we generated and characterized a novel BAC transgenic mouse line expressing GFP under the control of the AldoC promoter. Our findings demonstrate that AldoC BAC-GFP mice enable reliable and widespread labeling of astrocytes throughout the brain. This model overcomes several limitations of conventional astrocyte markers, such as GFAP, S100β, GLT-1, Aldh1L1, and Sox9, which exhibit variable expression or lack cell-type specificity in certain brain regions.

AldoC has been extensively studied in the cerebellum, particularly in Purkinje cells where it displays a distinctive striped pattern [25]. While this characteristic banding was observed in an isoform lobule of AldoC BAC-GFP mice, it was not apparent in the rostral cerebellum, suggesting region-specific regulatory differences. Notably, we also detected strong GFP expression in the cochlear nuclei and paraflocculus, indicating that AldoC BAC-GFP mice may have limited utility for studies requiring precise cerebellar stripe resolution, but remain informative for broader investigations of cerebellar astrocyte and Purkinje cell studies.

Importantly, AldoC BAC-GFP mice proved highly effective for labeling diverse astrocyte populations, including GFAP-negative astrocytes in the prefrontal cortex and striatum. The relatively low co-localization with S100β and GLT-1 in these regions further suggests the presence of astrocyte subtypes not adequately labeled by traditional markers. These results align with previous reports emphasizing the heterogeneity of astrocyte identity and density across brain regions. For example, we found that while cortical and striatal astrocyte densities were comparable, the hippocampus displayed ~ 30% higher astrocyte density (data not shown). Such observations underscore the value of AldoC BAC-GFP mice for comparative analyses of regional astrocyte populations.

Our present study demonstrates that AldoC promoter in the BAC-GFP mice drives GFP expression broadly in astrocytes across the brain. GFP signals are detected in a pan-astrocyte population with relatively low regional bias, allowing visualization of the global astrocyte network in vivo. Using astrocytic markers together enabled us to determine (1) whether AldoC BAC-GFP faithfully labels astrocytes, (2) which astrocyte subpopulations are included or excluded, and (3) how AldoC expression relates to established astrocyte markers in terms of functional and developmental characteristics. This approach is essential for demonstrating the suitability of the AldoC BAC-GFP mouse as a tool for studying astrocytes in both physiological and pathological contexts. Another strength of the AldoC BAC-GFP model is its capacity to support high-resolution morphological analyses without the need for exogenous labeling. Because BAC technology preserves endogenous regulatory elements, GFP expression is physiologically relevant and cell-type specific [26, 27]. This feature allows direct visualization of astrocyte morphology under diverse conditions, making the model well suited for studies of structural plasticity and reactive gliosis. Furthermore, GFP expression in Purkinje cells enables its application in investigation of cerebellar AldoC-expressing neuronal populations.

Beyond histological applications, AldoC BAC-GFP mice offer distinct advantages for functional and developmental studies. Conventional electrophysiological approaches often rely on fluorescent dyes (e.g., SR101 or OGB-1) to identify astrocytes, which can introduce technical variability or interfere with cellular physiology [28–30]. In contrast, AldoC-driven GFP expression allows for the direct targeting of astrocytes in slice recordings without exogenous labeling. This was validated by our findings that GFP-positive cells exhibited characteristic linear passive conductance, which was abolished by Spadin, a known TREK-1 potassium channel inhibitor. In addition, because BAC-GFP transgenic mice are powerful tools for studying gene expression during development [31], AldoC BAC-GFP mice can also be utilized to investigate the role of AldoC in brain development and serve as a valuable model for exploring astrocytic contributions during crucial developmental stages. Interestingly, a recently reported single-cell atlas of the developing human brain [32] revealed a biphasic expression of AldoC, showing a transient peak in radial glia and neuroblasts during the mid-fetal period (second trimester), followed by re-expression during adolescence (data not shown). Although these data were obtained from the developing human brain, they suggest that AldoC expression is under tight regulatory control. Therefore, examining AldoC expression using AldoC BAC-GFP mice will be essential for elucidating the role of AldoC during brain development.

In conclusion, the AldoC BAC-GFP transgenic mouse line represents a robust and versatile tool for astrocyte research. It enables reliable identification of astrocytes across diverse brain regions, supports both functional and morphological analyses, and provides new opportunities to investigate astrocyte heterogeneity under physiological and pathological conditions. We anticipate that this model will advance the understanding of astrocyte biology and contribute broadly to the field of glial neuroscience.

### Limitations and future perspectives

Despite these advantages, several limitations should be noted. First, AldoC expression is not uniformly present in all astrocyte populations, as evidenced by the absence of GFP labeling in the medial habenula. This underscores the need to consider regional heterogeneity when interpreting data from this model. Second, AldoC is also expressed in Purkinje cells, which, while offering opportunities for cerebellar studies, may confound strictly astrocyte-specific analyses in the cerebellum. Third, as with other BAC transgenic models, potential positional effects or copy number variation could influence transgene expression, warranting careful validation in different founder lines.

Future work should aim to combine the AldoC BAC-GFP line with additional genetic strategies, such as conditional reporters or intersectional labeling systems, to achieve even finer astrocyte subtype resolution. Integration with single-cell transcriptomics and spatial proteomics could further refine the identification of AldoC-positive astrocyte populations and clarify their roles in region-specific physiology and pathology. Finally, applying this model to disease contexts—such as epilepsy, neurodegeneration, or psychiatric disorders—may reveal new insights into astrocyte contributions to brain dysfunction.

## Supplementary Information

Below is the link to the electronic supplementary material.

Supplementary Material 1
Figure 1. Regional expression pattern of AldoC compared to canonical astrocytic markers. (A) Western blot analysis showing protein levels of AldoC, GFAP, and GLT-1 in the cortex, striatum, and hippocampus. β-tubulin was used as a loading control. (B) Quantification of protein expression levels normalized to β-tubulin (n = 4 per group). AldoC exhibited relatively uniform expression across regions, while GFAP and GLT-1 showed greater variability. Data are presented as mean ± SEM. (C) Representative low-magnification immunofluorescence image of a sagittal brain section stained with anti-AldoC antibody, showing broad expression throughout the brain. This image were acquired using a slide scanner. Insets (i–iii) correspond to magnified views of boxed regions in cortex, hippocampus, and cerebellum, respectively. These enlarged images were obtained using a confocal microscope in the same slice. Scale bars: main panel, 1000 μm; insets, 200 μm. (N_TG_ = 3, N_WT_ = 3).
Figure 2. Co-localization of GFP with AldoC and calbindin in the cerebellum of AldoC BAC-GFP transgenic mice. (A) Low-magnification image showing GFP (green) and AldoC (red) expression in the cerebellum. (B) High-magnification images (boxed region in A) show strong colocalization of GFP and AldoC in cerebellar lobules, including Purkinje cell layers. Left to right: GFP channel, AldoC channel, merged image. Scale bars: A, 200 μm; B, 100 μm. (C) Low-magnification image showing GFP (green) and calbindin (red) expression in the cerebellum. (D) High-magnification images (boxed region in C) demonstrate colocalization of GFP and calbindin in Purkinje cells. Left to right: GFP channel, calbindin channel, merged image. Scale bars: C, 200 μm; D, 100 μm. All images were acquired using a confocal microscope.
Figure 3. Region-specific distribution and morphological diversity of GFP-positive astrocytes in AldoC BAC-GFP transgenic mice. Representative fluorescence images showing GFP-expressing cells in various brain regions of AldoC BAC-GFP mice. Central sagittal brain overview highlights widespread GFP signal. Magnified views display region-specific astrocyte density and morphology in the cortex, corpus callosum, zona layer (zo) of the superior colliculus, olfactory bulb, hippocampal region, pons, and cerebellum. GFP-positive astrocytes exhibited radial morphologies in the cortex and olfactory bulb, while linear morphologies were more apparent in the cerebellum and pons. Astrocyte density was notably lower in white matter regions such as the corpus callosum. Scale bar: 50 μm. Macroscopic image was acquired using a slide scanner and high-resolution images were obtained using a confocal microscope in the same sample.
Figure 4. GFP-expressing cells co-localize with astrocytic markers Aldh1l1 and Sox9. (A–B) Representative confocal images and quantification pie charts showing the colocalization of GFP with Aldh1l1 and Sox9 in the prefrontal cortex (PFC), striatum, and hippocampal CA1 region. Scale bars: 100 µm (overview), 20 µm (magnified view). Arrowheads indicate double-labeled astrocytes. (N_TG_ = 3 (*n* = *6*), N_WT_ = 3 (*n* = *6*)).
Figure 5. Co-localization of NG2 with astrocytic markers in the hippocampus. (A) Representative immunofluorescence images showing co-staining of NG2 (green) and glutamine synthetase (GS, red) in the hippocampus. Merged image and enlarged view demonstrate partial overlap (arrowheads) between NG2-positive cells and GS. (B) Co-staining of NG2 (green) and GLT-1 (red) in the hippocampus. Merged image and enlarged region reveal co-localization (arrowheads), indicating potential expression of astrocytic markers in NG2-positive cells. Scale bars: 100 μm (left three panels), 50 μm (rightmost panels). (N_TG_ = 3, N_WT_ = 3).
Figure 6. Differential expression of astrocytic markers in the medial and lateral habenula. (A–C) Representative immunofluorescence images showing DAPI (blue), GFP (green), and astrocytic markers (red) in the medial habenula (mHb) and lateral habenula (lHb) of AldoC BAC-GFP mice. (A) Co-localization of GFP with glutamine synthetase (GS). (B) Co-localization of GFP with S100β. (C) Co-localization of GFP with GLT-1. Right panels show merged images. Yellow dashed lines delineate the mHb and lHb regions. (D-F) Quantification graphs of marker expression normalized to the mHb signal. GS and GLT-1 expression were significantly higher in the lHb, while S100β was more enriched in the mHb. Data are presented as mean ± SEM. Statistical significance was determined by unpaired *t*-test. *P* < 0.05; **P < 0.01; ***P < 0.001. Scale bars: 100 μm. (N_TG_ = 3 (*n* = *6*), N_WT_ = 3 (*n* = *6*)).

## Data Availability

No datasets were generated during the current study.
