# Supplementary material for: AldoC BAC-GFP transgenic mice as a reliable model for astrocyte identification and functional studies in the brain
Source: Mol Brain. 2025 Dec 4;19:2. doi: 10.1186/s13041-025-01264-0 (PMC12781538; doi:10.1186/s13041-025-01264-0)

Supplementary fig. 1

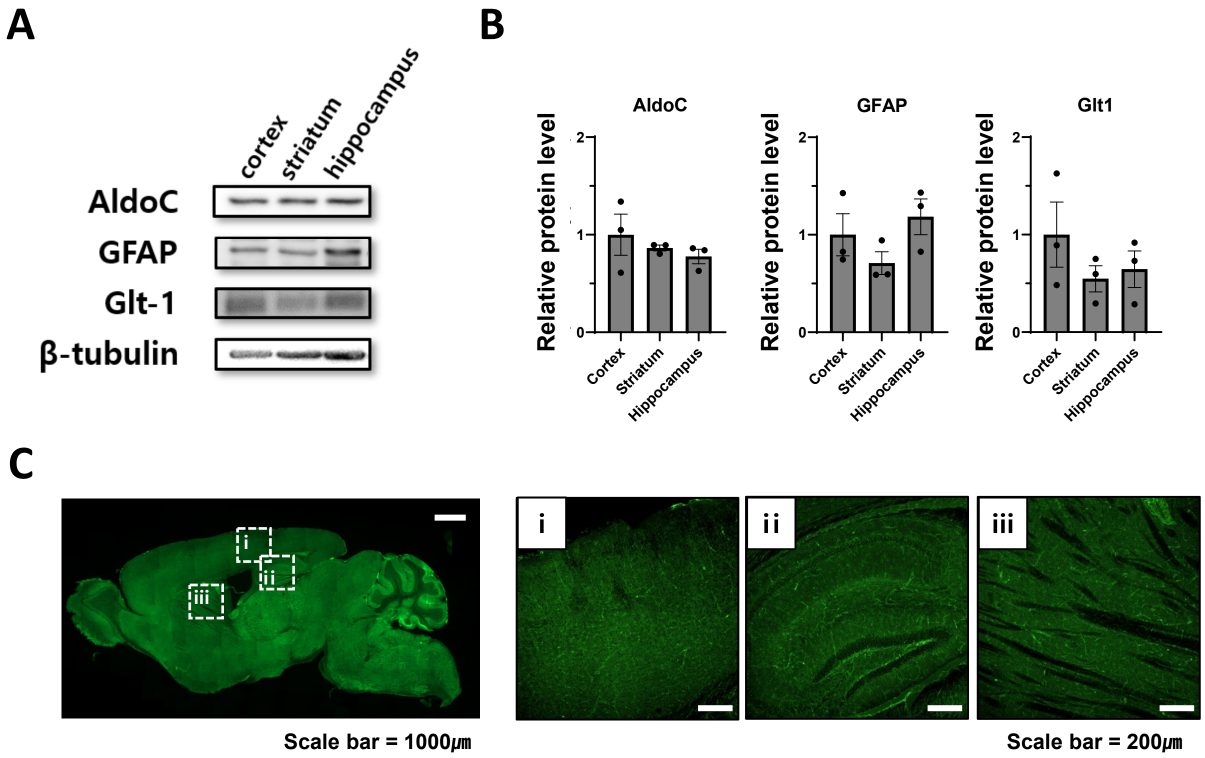

Supplementary fig. 2

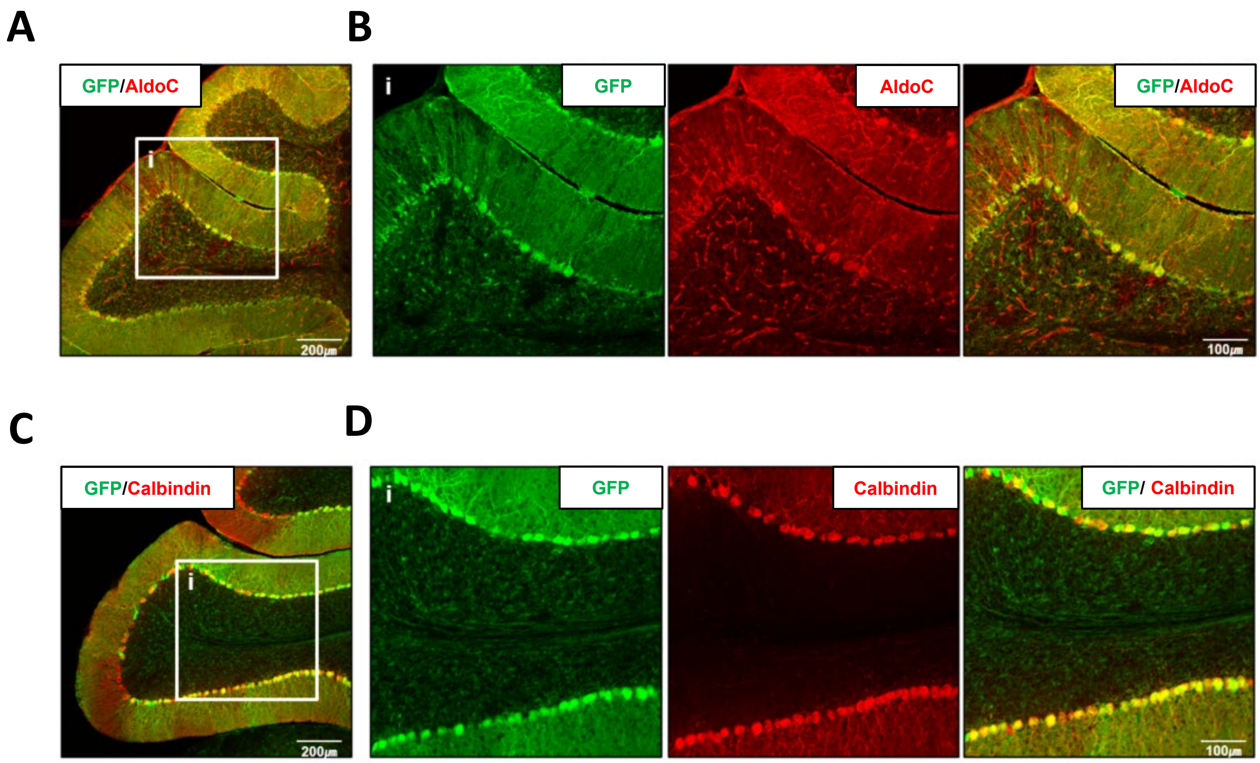

Supplementary fig. 3

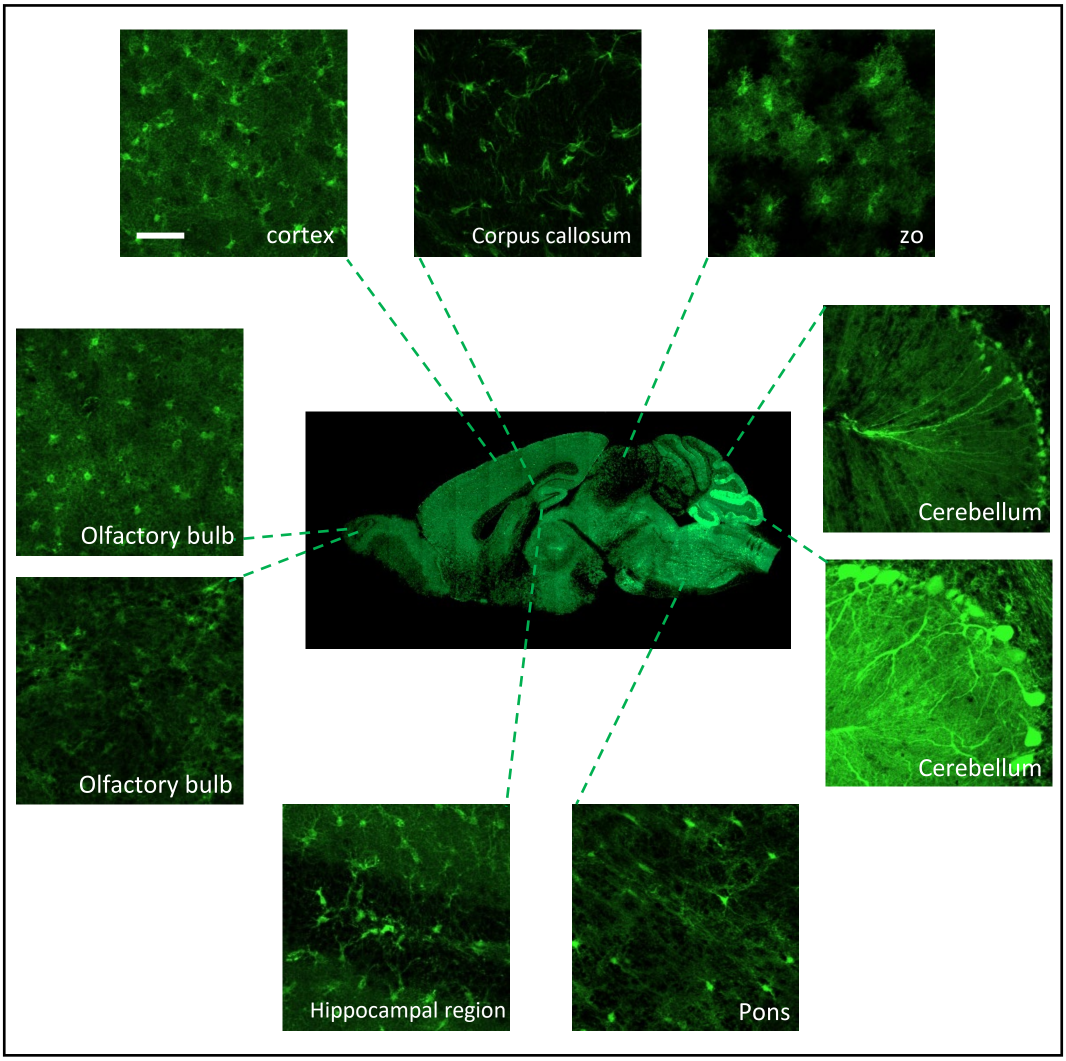

Scale bar = 50μm

Supplementary fig. 4

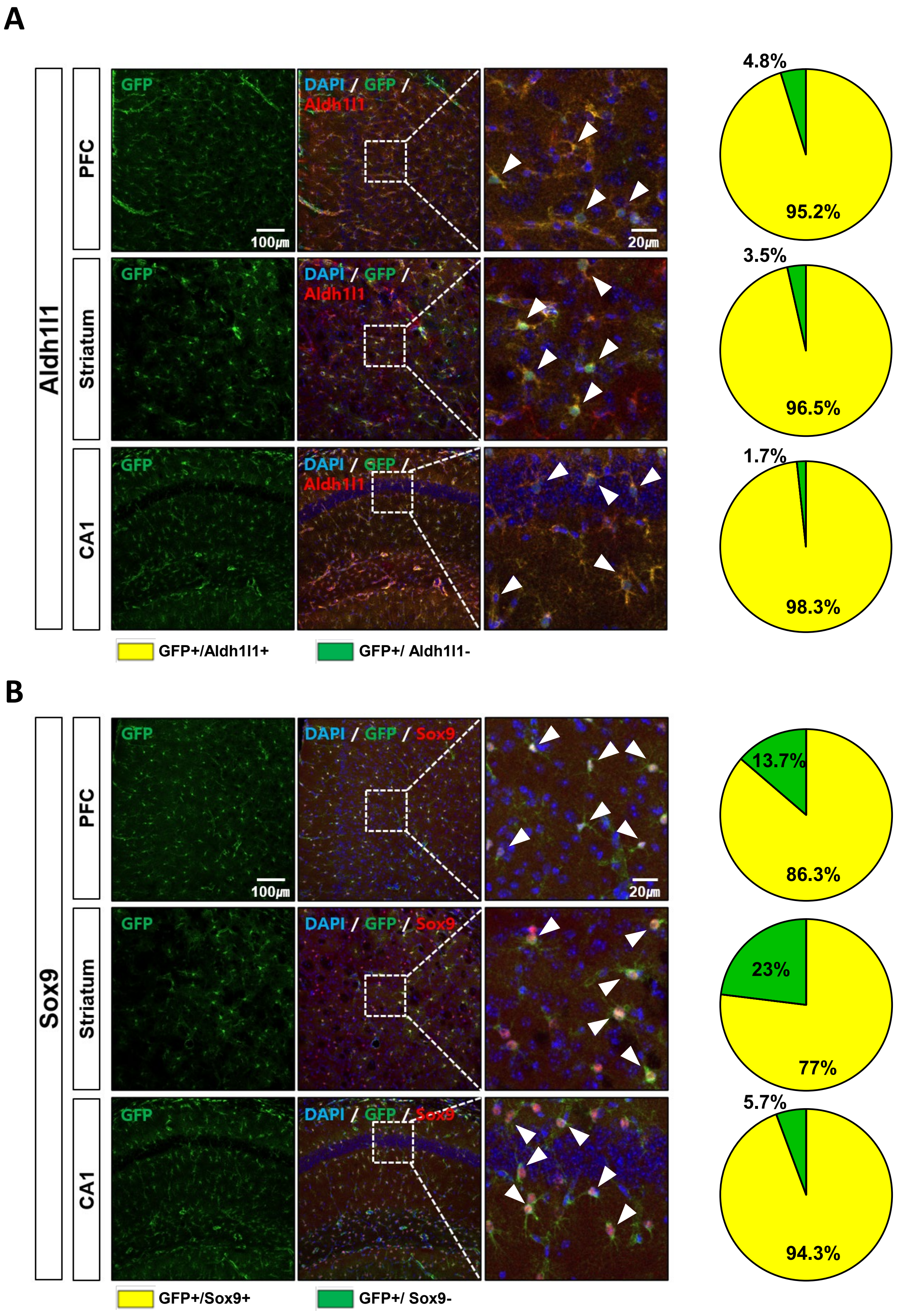

Supplementary fig. 5

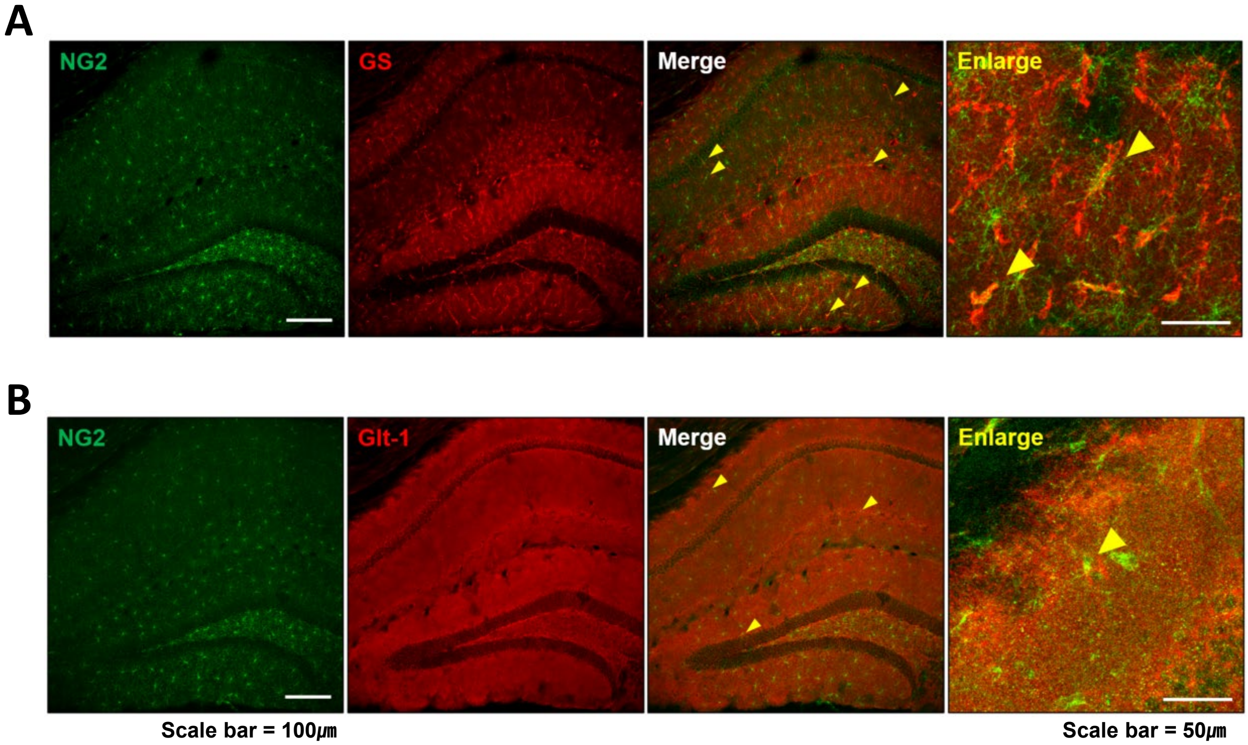

Supplementary fig. 6

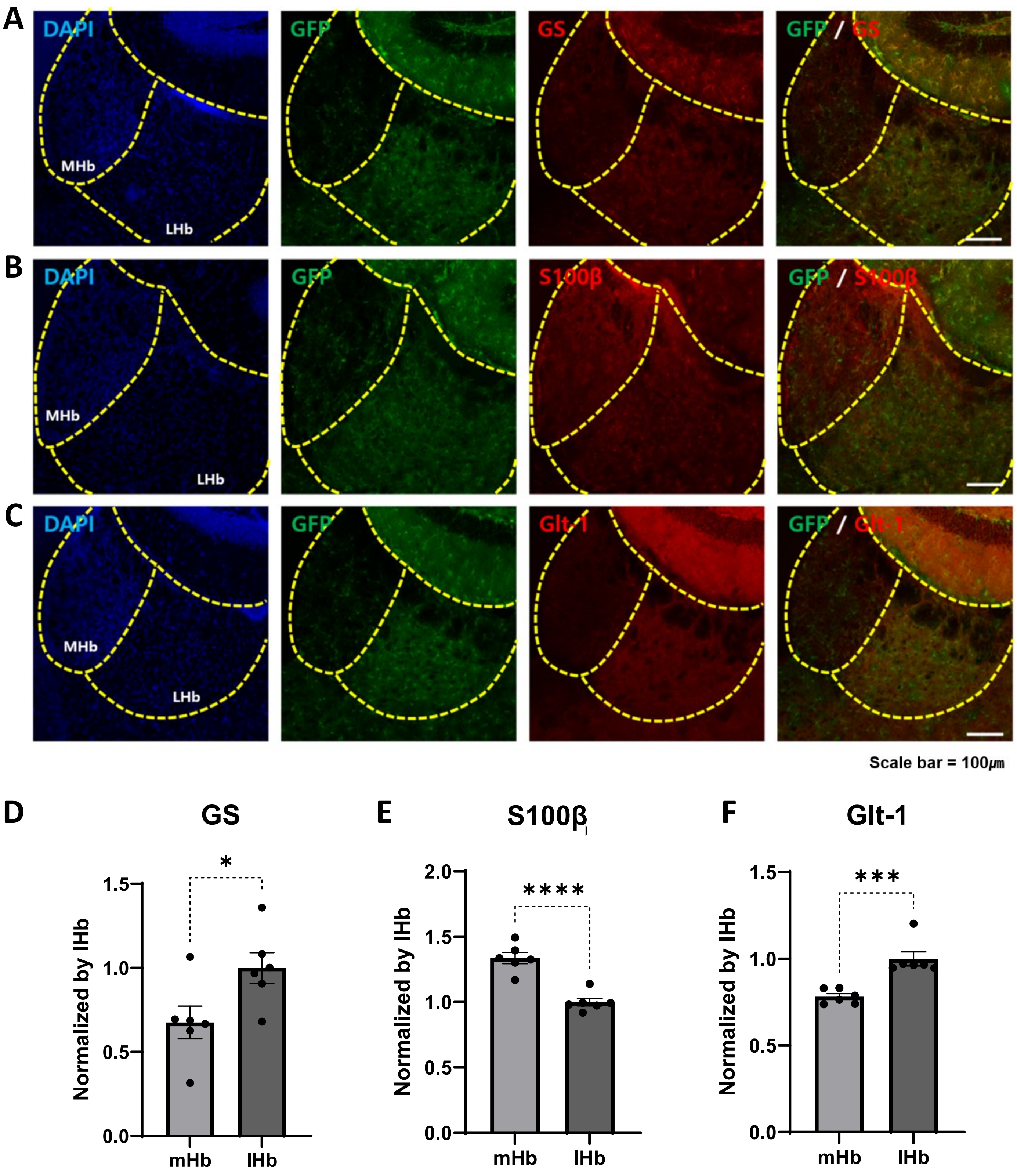

Supplement: Supplementary file 1 — Supplementary Material 1 Figure 1. Regional expression pattern of AldoC compared to canonical astrocytic markers. (A) Western blot analysis showing protein levels of AldoC, GFAP, and GLT-1 in the cortex, striatum, and hippocampus. β-tubulin was used as a loading control. (B) Quantification of protein expression levels normalized to β-tubulin (n = 4 per group). AldoC exhibited relatively uniform expression across regions, while GFAP and GLT-1 showed greater variability. Data are presented as mean ± SEM. (C) Representative low-magnification immunofluorescence image of a sagittal brain section stained with anti-AldoC antibody, showing broad expression throughout the brain. This image were acquired using a slide scanner. Insets (i–iii) correspond to magnified views of boxed regions in cortex, hippocampus, and cerebellum, respectively. These enlarged images were obtained using a confocal microscope in the same slice. Scale bars: main panel, 1000 μm; insets, 200 μm. (NTG = 3, NWT = 3). Figure 2. Co-localization of GFP with AldoC and calbindin in the cerebellum of AldoC BAC-GFP transgenic mice. (A) Low-magnification image showing GFP (green) and AldoC (red) expression in the cerebellum. (B) High-magnification images (boxed region in A) show strong colocalization of GFP and AldoC in cerebellar lobules, including Purkinje cell layers. Left to right: GFP channel, AldoC channel, merged image. Scale bars: A, 200 μm; B, 100 μm. (C) Low-magnification image showing GFP (green) and calbindin (red) expression in the cerebellum. (D) High-magnification images (boxed region in C) demonstrate colocalization of GFP and calbindin in Purkinje cells. Left to right: GFP channel, calbindin channel, merged image. Scale bars: C, 200 μm; D, 100 μm. All images were acquired using a confocal microscope. Figure 3. Region-specific distribution and morphological diversity of GFP-positive astrocytes in AldoC BAC-GFP transgenic mice. Representative fluorescence images showing GFP-expressing cells in va [file 13041_2025_1264_MOESM1_ESM.pdf]
